# Supplementary figures and images for: Development and characterization of 24 polymorphic microsatellite loci for the freshwater fish Ichthyoelephas longirostris (Characiformes: Prochilodontidae)
Source: PeerJ. 2016 Sep 1;4:e2419. doi: 10.7717/peerj.2419 (PMC5012415; doi:10.7717/peerj.2419)

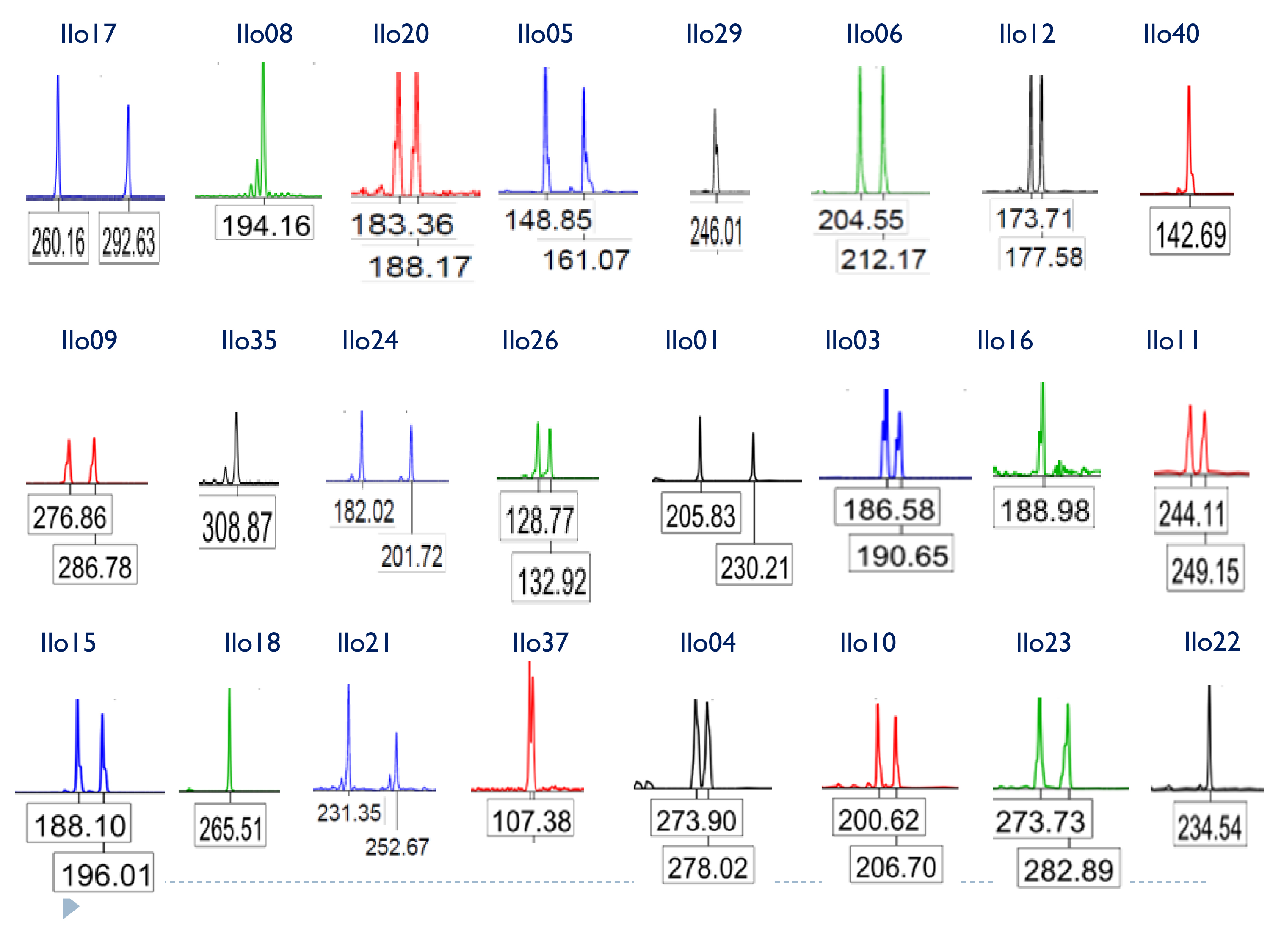

Supplement: Figure S1 [file peerj-04-2419-s001.jpg]
